# Uncovering the Mechanism of Drug Resistance Caused by the T790M Mutation in EGFR Kinase From Absolute Binding Free Energy Calculations

**DOI:** 10.3389/fmolb.2022.922839

**Published:** 2022-05-30

**Authors:** Huaxin Zhou, Haohao Fu, Han Liu, Xueguang Shao, Wensheng Cai

**Affiliations:** ^1^ Research Center for Analytical Sciences, Frontiers Science Center for New Organic Matter, College of Chemistry, Tianjin Key Laboratory of Biosensing and Molecular Recognition, State Key Laboratory of Medicinal Chemical Biology, Nankai University, Tianjin, China; ^2^ Haihe Laboratory of Sustainable Chemical Transformations, Tianjin, China

**Keywords:** absolute binding free energy calculation, Epidermal Growth Factor Receptor (EGFR), T790M mutation, drug resistance, molecular dynamics simulation, BFEE2

## Abstract

The emergence of drug resistance may increase the death rates in advanced non-small cell lung cancer (NSCLC) patients. The resistance of erlotinib, the effective first-line antitumor drug for NSCLC with the L858R mutation of epidermal growth factor receptor (EGFR), happens after the T790M mutation of EGFR, because this mutation causes the binding of adenosine triphosphate (ATP) to EGFR more favorable than erlotinib. However, the mechanism of the enhancement of the binding affinity of ATP to EGFR, which is of paramount importance for the development of new inhibitors, is still unclear. In this work, to explore the detailed mechanism of the drug resistance due to the T790M mutation, molecular dynamics simulations and absolute binding free energy calculations have been performed. The results show that the binding affinity of ATP with respect to the L858R/T790M mutant is higher compared with the L858R mutant, in good agreement with experiments. Further analysis demonstrates that the T790M mutation significantly changes the van der Waals interaction of ATP and the binding site. We also find that the favorable binding of ATP to the L858R/T790M mutant, compared with the L858R mutant, is due to a conformational change of the αC-helix, the A-loop and the P-loop of the latter induced by the T790M mutation. This change makes the interaction of ATP and P-loop, αC-helix in the L858R/T790M mutant higher than that in the L858R mutant, therefore increasing the binding affinity of ATP to EGFR. We believe the drug-resistance mechanism proposed in this study will provide valuable guidance for the design of drugs for NSCLC.

## Introduction

Lung cancer is the leading cause of cancer-related deaths worldwide (Jemal et al., 2011). The most common form of lung cancer is non-small cell lung cancer (NSCLC), which accounts for about 80–85% of lung cancer (Sharma et al., 2007; Inamura, 2017). In NSCLC, overexpression of epidermal growth factor receptor (EGFR) or hyper-activating mutations in its kinase domain have been observed in at least 50% of cases (Normanno et al., 2006). EGFR is a transmembrane receptor protein that has an essential role in cancer cell proliferation, survival, adhesion, migration, and differentiation by activating RAS/RAF/MEK/ERK and PI3K/AKT key downstream signaling pathways (Hirsch et al., 2003; Nagano et al., 2018; Zhou et al., 2022). In addition, among the currently marketed drugs, about 50–60% of drugs use membrane proteins to exert their effects (Santos et al., 2017). Therefore, EGFR and its mutations are one of the most valuable clinically validated drug targets for NSCLC treatment (Liao et al., 2010; Wee and Wang, 2017). A large number of small-molecule inhibitors acting on EGFR were developed to inhibit the kinase domain of EGFR and disrupt the oncogenic cell signaling by competing with adenosine triphosphate (ATP) for the binding site on the intracellular tyrosine kinase domain of EGFR. For example, first-generation EGFR inhibitor gefitinib or erlotinib is widely employed as first-line therapy for NSCLC with EGFR L858R mutation or exon 19 deletions. However, the secondary EGFR mutation T790M detected in NSCLC patients, can induce clinical resistance to gefitinib or erlotinib, greatly limiting the efficacy of these drugs in clinical use (Pao et al., 2005; Kosaka et al., 2006).

Understanding the mechanism of the T790M-induced drug resistance is important for further drug design. To this end, Kobayashi et al. proposed that the source of the acquired drug resistance was steric hindrance produced by the bulky methionine replaced the residue of threonine at position 790 (Kobayashi et al., 2005; Kwak et al., 2005; Pao et al., 2005). Interestingly, a later study demonstrated that the T790M resistance mutation increased the affinity of the receptor for ATP, which in turn diminished the potency of these ATP-competitive inhibitors (Yun et al., 2008). Several theoretical studies have been performed to explain the structural and energetic analyses of drug resistance conferred by the T790M mutation. Saldaña-Rivero and co-workers used the MM-GBSA approach to explain how L858R, T790M and L858R/T790M mutations impact the binding mechanism of ATP (Saldaña-Rivera et al., 2019). The popular MM/GBSA approach has been used to obtain a rough estimate of the binding free energy for a variety of complexes to explicate drug resistance (Zhang et al., 2019; Tan et al., 2022). A mechanistic explanation linking the mutations of the protein induce changes in the conformational free-energy landscape was also investigated by using massive molecular dynamics (MD) simulations together with parallel tempering, metadynamics, and one of the best force-fields available, showing a clear shift toward the active conformation for the T790M mutant and the L858R/T790M mutant (Sutto and Gervasio, 2013). The reason for the different binding affinities of ATP with respect to the L858R mutant and the L858R/T790M mutant, however, is still unclear. In addition, the relationship of the conformation changes of A-loop, αC-helix and P-loop and the difference of binding affinity remains to be further explored.

In this article, the standard binding free energies of ATP with respect to two EGFR mutants (L858R, L858R/T790M) have been calculated to investigate the mechanism of the drug resistance induced by the T790M mutation. Pair interaction calculations have been performed to reveal the driving force underlying the change of binding affinity of ATP to EGFR due to the T790M mutation and structural analysis has been carried out to capture the conformational change of the complex. The present study shows the essential reason for the drug resistance induced by the T790M mutation, which can provide useful guidance for the further drug design against drug resistance.

## Methods

### Structural Modeling

As the cocrystallized structure of EGFR or its mutants in a complex with ATP has not yet been solved, here, we adopted nonhydrolyzable analog AMP of ATP to carry out this research. The crystal structure of an EGFR L858R mutant kinase domain bound with the AMP molecule (PDB: 2EB3) as the structure template to model the EGFR L858R/T790M-AMP complex by CHARMM-GUI (Jo et al., 2008). Neither the protein nor the ligand was protonated. Missing residues in the retrieved structures were also examined and reconstructed using CHARMM-GUI. The atomic coordinates of the EGFR conformations were obtained from the Protein Data Bank (PDB) (http://www.pdb.org).

### Molecular Dynamics Simulations

MD simulations for all EGFR models were performed using explicit-solvent periodic boundary conditions using NAMD (Phillips et al., 2020). Each model was solvated in a cubic box of TIP3P water, keeping a distance of 15 Å between the protein and the sides of the solvent box (Jorgensen et al., 1983). Each of the solvated systems was neutralized by adding enough chloride and sodium ions to give a concentration of 250 nM. The CHARMM36m protein force field was used to simulate all protein structures (Huang et al., 2017). The CHARMM General Force Field (CGenFF) force field was used to model the organic molecules (Vanommeslaeghe et al., 2010). All heavy atoms were restrained at the first stage of minimization. After that, the heavy atoms of ligand were fixed in the second step. Finally, all atoms in the system were minimized without any restraint. Production simulations were subsequently performed under the NPT condition at 300 K and 1.013 bar of the system. Temperature and pressure were held constant using Langevin dynamics and the Langevin piston (Uhlenbeck and Ornstein, 1930; Feller et al., 1995). All the trajectories were visualized using the VMD software (Humphrey et al., 1996).

### Calculation of Standard Binding Free Energy

The binding free energy acts as a useful index to evaluate the binding affinity between mutants and drugs, and can be used as an important indicator of drug resistance (Zhou et al., 2013; Ma et al., 2015; Khan et al., 2020). In this article, the standard binding free-energy calculations of all systems were performed employing BFEE2 and following a geometrical route (Gumbart et al., 2013; Fu et al., 2021; Fu et al., 2022). BFEE2, which is a graphical user interface-based software, can automatically set up and analyze absolute binding free-energy calculations carried out with the popular MD engine NAMD (Fu et al., 2021; Fu et al., 2022). The calculation process of each protein-ligand complex was divided into eight independent subprocesses. Seven collective variables of geometrical restraints, that is, the root-mean-square deviation (RMSD) for describing the conformational change of the ligand in its bound state with respect to its native conformation, three Euler angles (*Θ*, *Φ*, *Ψ*) for describing the relative orientation of the ligand, the polar and azimuthal angles (*θ*, *φ*), together with the distance (*r*) between the center of mass of the ligand and that of the protein for describing its relative position (Fu et al., 2017), were introduced to accelerate the convergence of free-energy calculations. The contributions of the geometric restraints were evaluated by means of one-dimensional potential of mean force calculations carried out using the well-tempered meta-eABF (WTM-ABF) algorithm (Fu et al., 2016; Lesage et al., 2017; Fu et al., 2018; Fu et al., 2019).

## Results and Discussion

### Structural Analysis of Ligand-Protein Complexes

**FIGURE 1:**
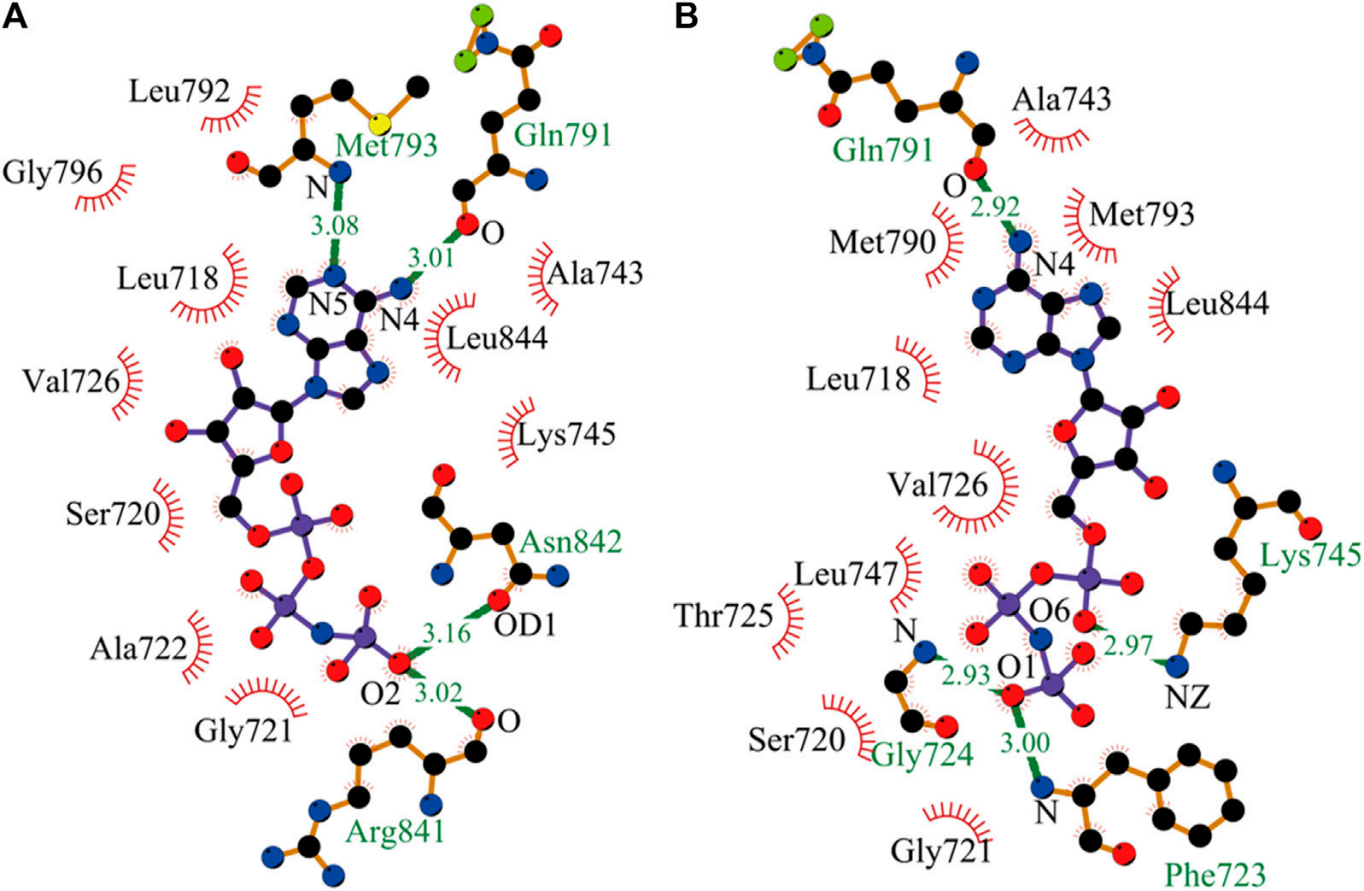
Interactions of AMP with its nearby residues in EGFR mutants, plotted by LIGPLOT. **(A)** Analysis of hydrogen bond and hydrophobic interaction of binding interface in the L858R mutant. **(B)** The L858R/T790M mutant (Wallace et al., 1995; Laskowski and Swindells, 2011).

Here, after the equilibrated simulations of all systems were completed, the intermolecular interactions of AMP with EGFR mutants were analyzed by the LIGPLOT program. As shown in Figure 1, AMP forms four specific hydrogen bonds with kinase polar residues Gln791, Met793, Arg841, and Asn842 of the L858R mutant and presents a wide hydrophobic contact interface with a number of kinase nonpolar residues, Leu792, Gly796, Val726, Leu718, Ser720, Ala722, Gly721, Ala743, Leu844, and Lys745. Interestingly, AMP also forms four hydrogen bonds with the L858R/T790M mutant, with an average distance shorter than those formed between AMP and the L858R mutant. However, these structural results may not completely explain the experimental observation from kinase assays that AMP has a higher binding affinity with the L858R/T790M mutant compared to the L858R one.

### Absolute Binding Free Energy of AMP to EGFR Mutants

**FIGURE 2:**
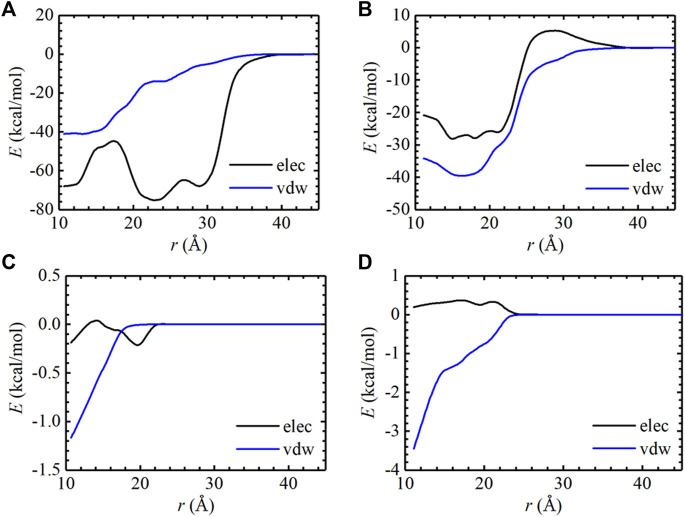
Pair interaction energy for the separation of the L858R mutant: AMP **(A)** and the L858R/T790M mutant: AMP **(B)** were decoupled into electrostatic and van der Waals contributions. The pair interaction energy for the separation of the Thr790 residue: AMP **(C)** and the Met790 residue: AMP **(D)** were decoupled into electrostatic and van der Waals contributions.

To evaluate the binding affinity of AMP with EGFR mutants, standard binding free-energy calculations were carried out on two complexes, i.e., AMP-L858R and AMP-L858R/T790M using the CHARMM36m force fields. The computed binding free-energy between AMP and EGFR kinase domains, with the contributions of geometric restraints acting on each degree of freedom, are reported in Table 1. The calculated standard binding free energies of AMP with respect to the L858R and the L858R/T790M double mutant are −5.69 kcal/mol and −6.72 kcal/mol, respectively. These estimates are in good agreement with the experimental values, namely, −5.25 kcal/mol and −6.96 kcal/mol, respectively, suggestive of a remarkable accuracy of BFEE2-based streamlined free-energy calculations. As expected, the binding affinity of AMP to EGFR increased by approximately 1.03 kcal/mol due to the T790M mutation. This result explains that the T790M substitution confers resistance by increasing the affinity for ATP, which was also demonstrated by (Yun et al., 2008). The one-dimensional free-energy profiles for the different contributions are presented in Supplementary Figures S1, S2. As described in Table 1, the major contribution of the absolute binding free energies of AMP with the L858R mutant and L858R/T790M mutant was the -1/*β* ln (S*I*C ^°^) term in Table 1, which characterizes the separation of the protein and the ligand. The pair interaction energy for the separation was further decoupled into the van der Waals and electrostatic terms, as depicted in Figures 2A,B. It is apparent that electrostatic interactions constitute the driving force for the binding of AMP to the L858R mutant. Both van der Waals and electrostatic interactions, however, are critical to the binding of AMP to the L858R/T790M mutant. In addition, the energy profile characterizing AMP and residue 790 was analyzed. As shown in Figures 2C,D, the T790M mutation increases van der Waals interactions of AMP to EGFR. Based on these results, we conclude that the higher binding affinity of AMP to the L858R/T790M mutant, compared to the L858R one, probably because the T790M mutation increases the van der Waals interaction between AMP and EGFR.

**TABLE 1:** Absolute binding free energies (in kcal/mol) for the ligand to EGFR mutants.

Contribution	L858R	Simulation time (ns)	L858R/T790M	Simulation time (ns)
$△G_{c}^{\mathrm{site}}$	−9.52 ± 0.66	20	−9.57 ± 0.28	30
$△G_{\Theta }^{\mathrm{site}}$	−0.58 ± 0.07	10	−0.42 ± 0.04	30
$△G_{\Phi }^{\mathrm{site}}$	−0.40 ± 0.04	20	−0.48 ± 0.08	30
$△G_{\Psi }^{\mathrm{site}}$	−0.35 ± 0.02	10	−0.45 ± 0.07	30
$△G_{\theta }^{\mathrm{site}}$	−0.11 ± 0.02	30	−0.23 ± 0.04	30
$△G_{\varphi }^{\mathrm{site}}$	−0.13 ± 0.01	30	−0.17 ± 0.02	30
$-\frac{1}{\beta }\ln\left(S*I*C^{\circ }\right)$	−11.01 ± 0.38	530	−10.43 ± 0.96	500
$△G_{c}^{\mathrm{bulk}}$	9.77 ± 0.11	20	8.36 ± 0.32	30
$△G_{o}^{\mathrm{bulk}}$	6.63	-	6.67	-
$△G_{\mathrm{bind}}^{o}$	−5.69 ± 0.48	670	−6.72 ± 0.91	710
△*G* ^o^_bind_ (exp)^a^	−5.25	-	−6.96	-

a Experimental binding free energies [△*G* ^o^_bind_(exp)^a^] for L858R and L858R/T790M come from (Yun et al., 2008).

### Analysis of the Structural Conformational Changes Underlying the Increase of Binding Free Energy

**FIGURE 3:**
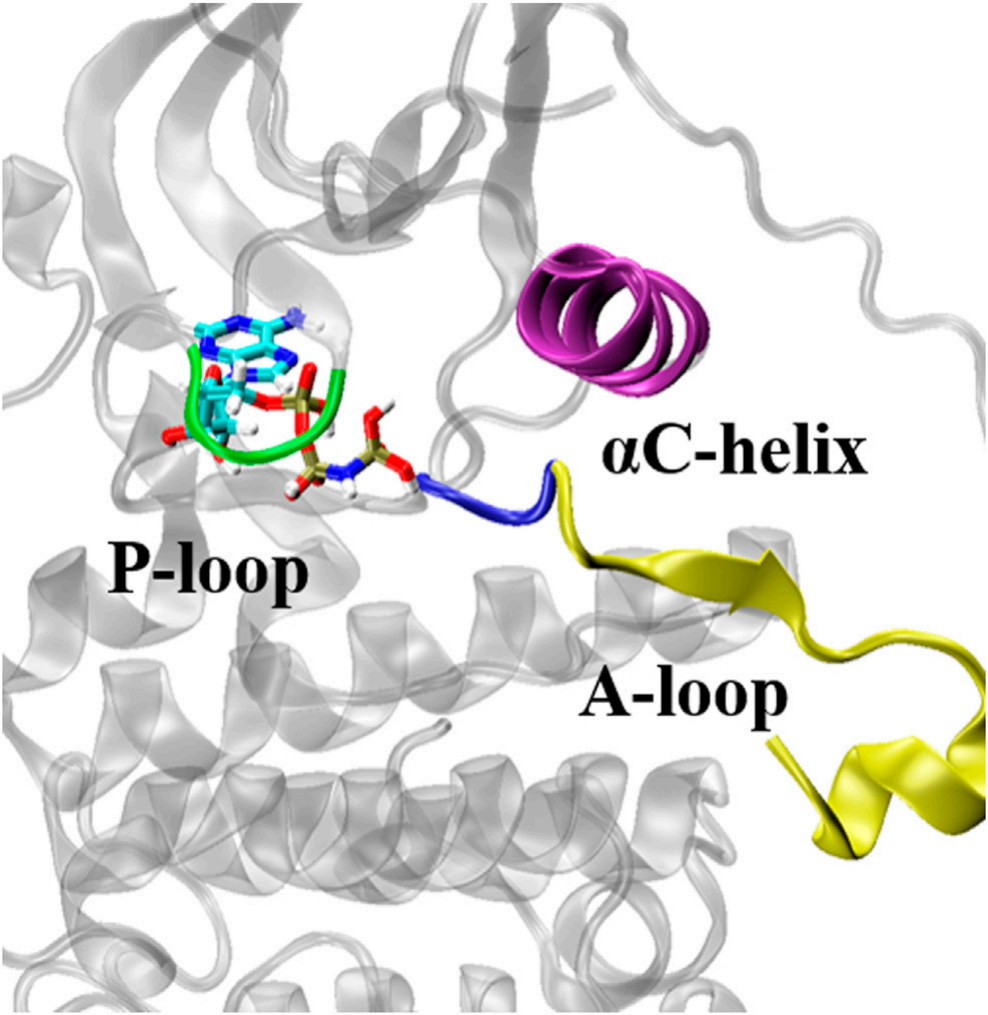
Key structural elements structures of EGFR. The key structural elements are highlighted in purple (αC helix), yellow (A-loop), blue (the Asp-Phe-Gly motif) and green (P-loop).

**FIGURE 4:**
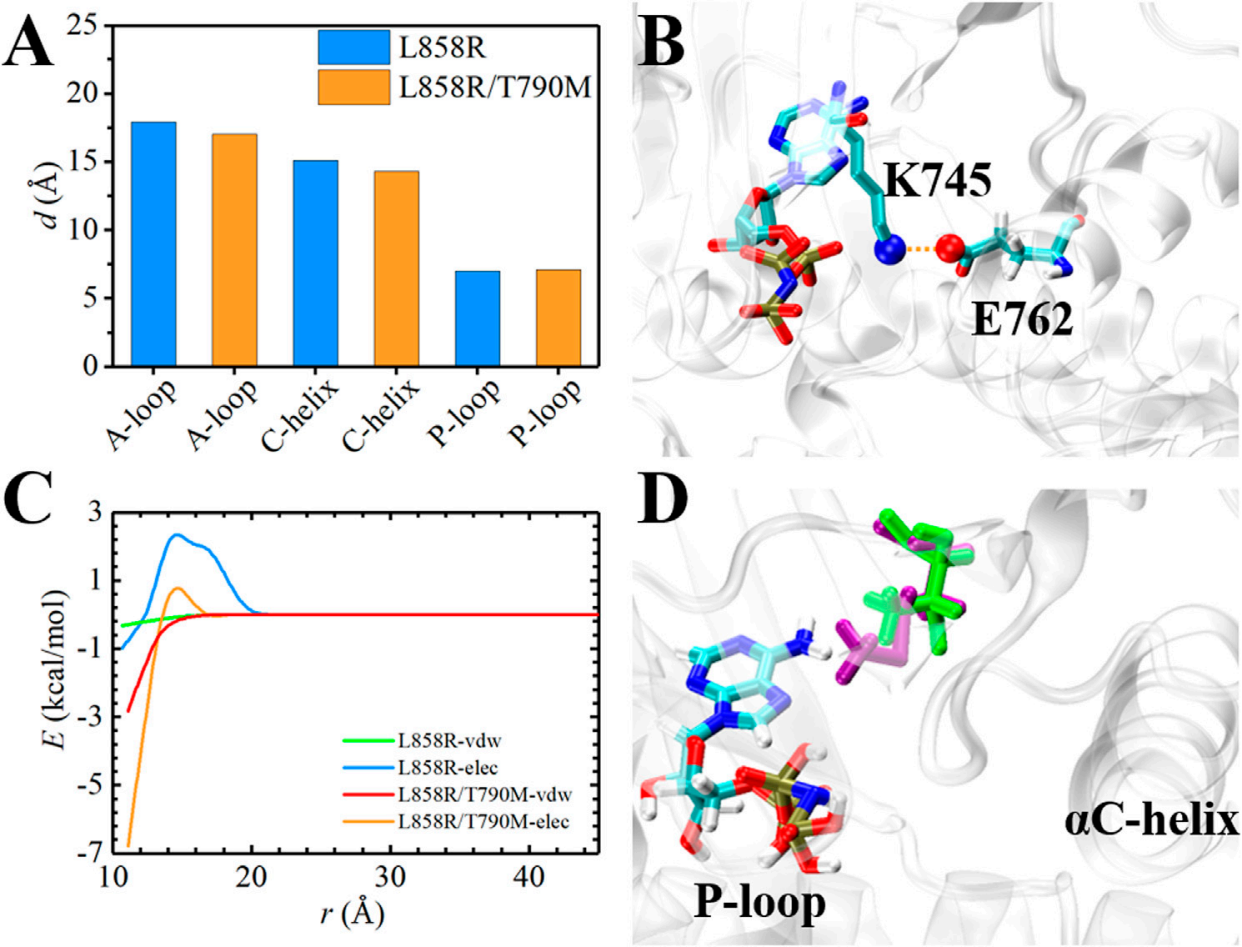
Structural analysis of EGFR mutants and AMP. **(A)** Time-evolution of the average distance between A-loop, αC-helix and P-loop and AMP, respectively. **(B)** The interaction of E762 and K745 in L858R/T790M mutant. **(C)** The pair interaction energy for the separation between the ligand and αC-helix of the EGFR mutants. **(D)** Superposition and comparison between the structures of Thr790-AMP pair and mutant Met790-AMP pair. Thr790 and Met790 are colored in green and purple, respectively.

The ATP-binding pocket is composed of a hinge region, A-loop, αC-helix, and P-loop (Figure 3), which are known to be crucial for their conformational stabilities and functional interactions with ATP (Johnson et al., 1996). The conformational changes of A-loop, P-loop, and αC-helix are important events occurring during kinase activation. In this section, we investigated the relationship between the structural changes of these key elements and binding affinity. We characterized the conformational changes of these key elements of EGFR by measuring the distance between these critical elements and ligand (Hu et al., 2022). As shown in Figure 4A, the location of AMP relative to A-loop, αC-helix and P-loop have a shorter distance in the L858R/T790M mutant, contributing to the favorable interactions that existed in the complex. Moreover, Figure 4B shows that the αC-helix is kept in place by a salt bridge formed by E762 and K745 in the L858R/T790M mutant, which is more stable than that observed in the L858R mutant (the average distance between N2 (Lys745) and CD (Glu762) of 3.05 Å vs. 7.86 Å, respectively). Additionally, the pair interaction energy for the separation was further decoupled into the van der Waals and electrostatic terms. As can be seen in Figure 4C, electrostatic interactions constitute the driving force for the binding of αC-helix to AMP in the L858R mutant. Although both electrostatic interactions and van der Waals interactions contribute to the binding of αC-helix to AMP in the L858R/T790M mutant, it is apparent that the effects of electrostatic interactions in higher than van der Waals interactions. The interactions of AMP and A-loop and P-loop are provided in Supplementary Figure S3. Further analysis revealed that the Met790 residue possesses a longer side chain that can has a favorable contact with AMP compared with the Thr790 residue during the conformational change process (Figure 4D). This phenomenon is in agreement with the results of Figures 2C,D. Based on the discussion above, after the T790M mutation, the structural changes of αC-helix and P-loop mainly improve electrostatic interactions and van der Waals interactions, respectively. These are profitable to the binding affinity of AMP to EGFR.

## Conclusion

Here, a powerful tool, BFEE2, was used to calculate the standard binding free energies of AMP to EGFR mutants. The results are well-consistent with the experiment. We found that the kinase affinity for AMP increased after the T790M mutation. In addition, our results indicate that electrostatic interaction plays a leading role in the binding of AMP to the L858R mutant, while both electrostatic interaction and van der Waals interaction are equally important for the binding of AMP to the L858R/T790M mutant. The present work emphasizes that the increased affinity of AMP to the L858R/T790M mutant compared with the L858R mutant is due to better stabilization of the active state for the mutant. This change may increase the interactions of AMP and P-loop, αC helix after the T790M mutation, therefore enhancing the binding affinity of AMP to EGFR. Although the calculated standard binding free energies are in good agreement with experimental values, there are challenges in the calculation of the standard binding free energies of EGFR inhibitors, especially for some of the fourth generation EGFR inhibitors without accurate binding sites. Still, the present work offers a perspective of the binding affinity of AMP to EGFR mutants and opens an avenue for further exploration of anticancer drugs acting on the EGFR to overcome drug resistance caused by the T790M mutation.

## Data Availability

The original contributions presented in the study are included in the article/Supplementary Material, further inquiries can be directed to the corresponding authors.
