# Supplementary material for: Uncovering the Mechanism of Drug Resistance Caused by the T790M Mutation in EGFR Kinase From Absolute Binding Free Energy Calculations
Source: Front Mol Biosci. 2022 May 30;9:922839. doi: 10.3389/fmolb.2022.922839 (PMC9189374; doi:10.3389/fmolb.2022.922839)
Supplement: Supplementary file 1 [file DataSheet1.PDF]

Supplementary Material: Uncovering the mechanism of drug resistance caused by the T790M mutation in EGFR kinase from absolute binding free energy calculations

Huaxin Zhou[‡,§], Haohao Fu[‡,§], Han Liu[‡,§], Xueguang Shao[*,‡,§], Wensheng Cai[*,‡,§]

[‡]Research Center for Analytical Sciences, Frontiers Science Center for New Organic Matter, College of Chemistry, Tianjin Key Laboratory of Biosensing and Molecular Recognition, State Key Laboratory of Medicinal Chemical Biology, Nankai University, Tianjin 300071, China

[§]Haihe Laboratory of Sustainable Chemical Transformations, Tianjin 300192, China

***Corresponding Author**: xshao@nankai.edu.cn, wscai@nankai.edu.cn

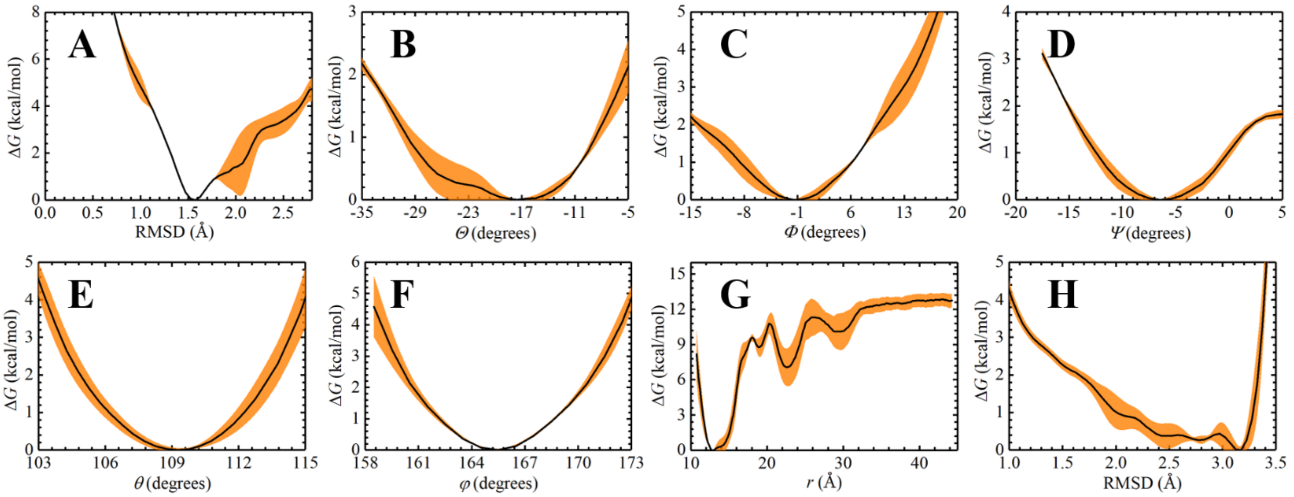

Supplementary Figure 1. PMF corresponding to each contribution of the L858R mutant determined with the CHARMM36m force field. Plots for the RMSD in the bound state and in the bulk in A and H, for the three Euler angles, Θ, Φ, and Ψ are given in B-D, respectively, for the positional restraints on θ and φ in E and F, for the separation in G. The error bars are showed in orange.

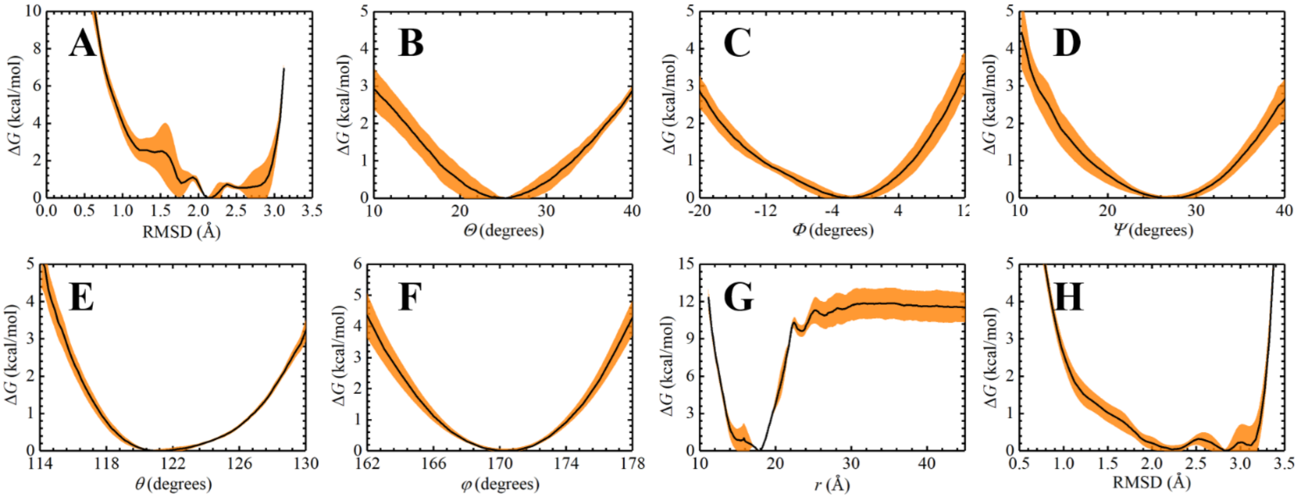

Supplementary Figure 2. PMF corresponding to each contribution of the L858R/T790M mutant determined with the CHARMM36m force field. Plots for the RMSD in the bound state and in the bulk in A and H, for the three Euler angles, Θ, Φ, and Ψ are given in B-D, respectively, for the positional restraints on θ and φ in E and F, for the separation in G. The error bars are showed in orange.

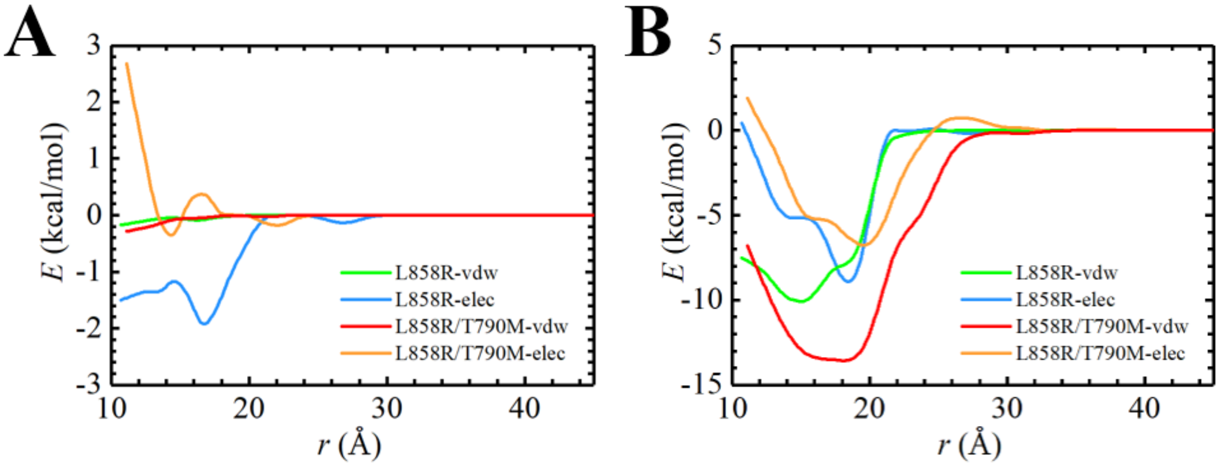

Supplementary Figure 3. Pair interaction energies for the separation between the ligand and A-loop, P-loop of the EGFR mutants. (A) A-loop. (B) P-loop.
